# Association between changes in estradiol, serotonin, and BDNF and antidepressant response in perimenopausal depression: a retrospective cohort study comparing integrated TCM-acupuncture with SSRIs

**DOI:** 10.3389/fpsyt.2026.1826598

**Published:** 2026-07-15

**Authors:** Jian Peng, Xiaoli Gan, Xiong Li

**Affiliations:** 1 Department of Traditional Chinese Medicine, Pingxiang Maternity and Child Health Care Hospital, Pingxiang, Jiangxi, China; 2 Laboratory Department, Pingxiang Maternity and Child Health Care Hospital, Pingxiang, Jiangxi, China

**Keywords:** acupuncture, BDNF, estradiol, HAMD-17, multivariable-adjusted cohort, perimenopausal depression, serotonin, traditional Chinese medicine

## Abstract

**Background:**

Perimenopausal depression (PMD) involves complex neuroendocrine interactions among estradiol (E_2_), serotonin (5-HT), and brain-derived neurotrophic factor (BDNF). Integrated traditional Chinese medicine and acupuncture (TCM-Acu) is widely used clinically, but its biological mechanisms relative to selective serotonin reuptake inhibitors (SSRIs) remain poorly characterized.

**Methods:**

We conducted a single-center retrospective cohort study in 240 perimenopausal women (TCM-Acu, n = 120; SSRIs, n = 120) treated for a minimum of eight weeks. Treatment effects were estimated using multivariable-adjusted regression (ANCOVA) accounting for baseline imbalances in age, disease duration, HAMD-17 score, and other covariates. Biomarker associations were examined via treatment × ΔBiomarker interaction terms.

**Results:**

In multivariable-adjusted analyses, TCM-Acu was associated with significantly greater HAMD-17 improvement relative to SSRIs (adjusted β = 3.79 points, 95% CI 2.79–4.79; P < 0.001; unadjusted mean change: 7.07 vs. 3.24 points). However, absolute treatment response rates (≥50% HAMD-17 reduction) were low in both groups (14.2% vs. 5.0%), indicating the observed difference reflects a relative rather than absolute clinical advantage. TCM-Acu was also associated with greater increases in 5-HT (adjusted β = 9.24 ng/mL, 95% CI 6.92–11.56; P < 0.001) and BDNF (adjusted β = 1.60 ng/mL, 95% CI 0.95–2.25; P < 0.001). Unadjusted estradiol levels did not differ significantly between groups (P = 0.592). Across the full cohort, changes in 5-HT and BDNF were positively associated with HAMD-17 improvement (Spearman ρ = 0.31 and 0.37, respectively), with the BDNF–HAMD-17 association being more pronounced in the TCM-Acu group on interaction testing.

**Conclusion:**

In multivariable-adjusted analyses, TCM-Acu was associated with greater HAMD-17 improvement and more pronounced increases in 5-HT and BDNF compared to SSRIs, while both treatments showed minimal differential effects on estradiol. However, absolute response rates were low in both groups, and the between-group difference reflects a relative rather than absolute clinical advantage. These observational findings are consistent with—but do not establish—the hypothesis that TCM-Acu’s antidepressant associations may be mediated primarily through serotonergic and neurotrophic pathways; this requires prospective confirmation. Key limitations include the retrospective single-center design, baseline imbalances, absence of a sham acupuncture control, and differential treatment durations, which collectively preclude causal inference.

## 1 Introduction

Perimenopause represents a critical window of heightened vulnerability to depressive disorders in women. Epidemiological evidence consistently indicates that the risk of incident depression increases two- to threefold during the menopausal transition compared to the premenopausal period (1, 2). The Study of Women’s Health Across the Nation (SWAN) demonstrated that perimenopausal women had significantly elevated odds of a major depressive episode relative to their premenopausal counterparts, independent of prior depressive history (3). In China, the incidence of depressive symptoms among perimenopausal women is estimated to range from 26% to 39%, imposing a substantial burden on both individuals and the healthcare system (4). Menopausal symptom burden also varies across perimenopausal substages and is associated with psychosocial factors such as social support and resilience (5). Current clinical guidelines recommend evidence-based antidepressant treatment, including selective serotonin reuptake inhibitors (SSRIs), for PMD, either as monotherapy or in combination with hormone therapy when clinically indicated (6–8). However, SSRIs are associated with adverse effects including sexual dysfunction, sleep disturbance, and weight gain, and a meaningful proportion of patients fail to achieve adequate clinical response, underscoring the need for effective alternatives (9).

The pathophysiology of PMD is increasingly understood as the convergence of three interacting biological axes: the hypothalamic–pituitary–gonadal (HPG) axis, the serotonergic system, and the neurotrophic signaling pathway. Declining estradiol (E_2_) and estrogen fluctuations during perimenopause are thought to reduce serotonin biosynthesis and transporter expression via estrogen receptor beta (ERβ)-mediated transcriptional regulation in limbic and hippocampal circuits (10–15). ERβ additionally upregulates brain-derived neurotrophic factor (BDNF) and its high-affinity receptor tropomyosin receptor kinase B (TrkB) in the hippocampus; estrogen withdrawal therefore precipitates both serotonergic deficiency and impaired neuroplasticity, both of which are established substrates of depression (16, 17). Consistent with this model, serum BDNF levels are significantly lower in perimenopausal women with depression compared to non-depressed controls, and correlate inversely with HAMD-17 scores (18). Despite theoretical coherence, clinical trials have produced inconsistent findings: some RCTs report that acupuncture improves depressive symptoms without significantly altering E_2_, follicle-stimulating hormone (FSH), or luteinizing hormone (LH) concentrations, suggesting that neurochemical and hormonal mechanisms may be partially dissociated *in vivo* (19, 20).

Integrated TCM-acupuncture--combining standardized Chinese herbal formulae with structured acupuncture protocols--has been evaluated in systematic reviews, meta-analyses, and trial protocols (19, 21–24), with related randomized and feasibility trials reporting effects of acupuncture or Chinese medicine interventions for perimenopausal depression and associated symptoms (20, 25–27). However, the majority of existing evidence focuses on symptom-level outcomes and does not systematically characterize the differential effects of TCM-Acu versus SSRIs on the E_2_–5-HT–BDNF axis. The mechanism by which TCM-Acu achieves antidepressant effects—whether through hormonal restoration, serotonergic upregulation, neurotrophic enhancement, or a combination thereof—remains an open question with direct implications for treatment selection and personalization. To address this gap, we conducted a retrospective cohort study comparing TCM-Acu with SSRIs in perimenopausal women, with the primary objective of characterizing the associations between treatment-induced changes in E_2_, 5-HT, and BDNF and antidepressant response, and determining whether these associations differ systematically between the two treatment modalities.

## 2 Methods

### 2.1 Study design and participants

**Figure 1:**
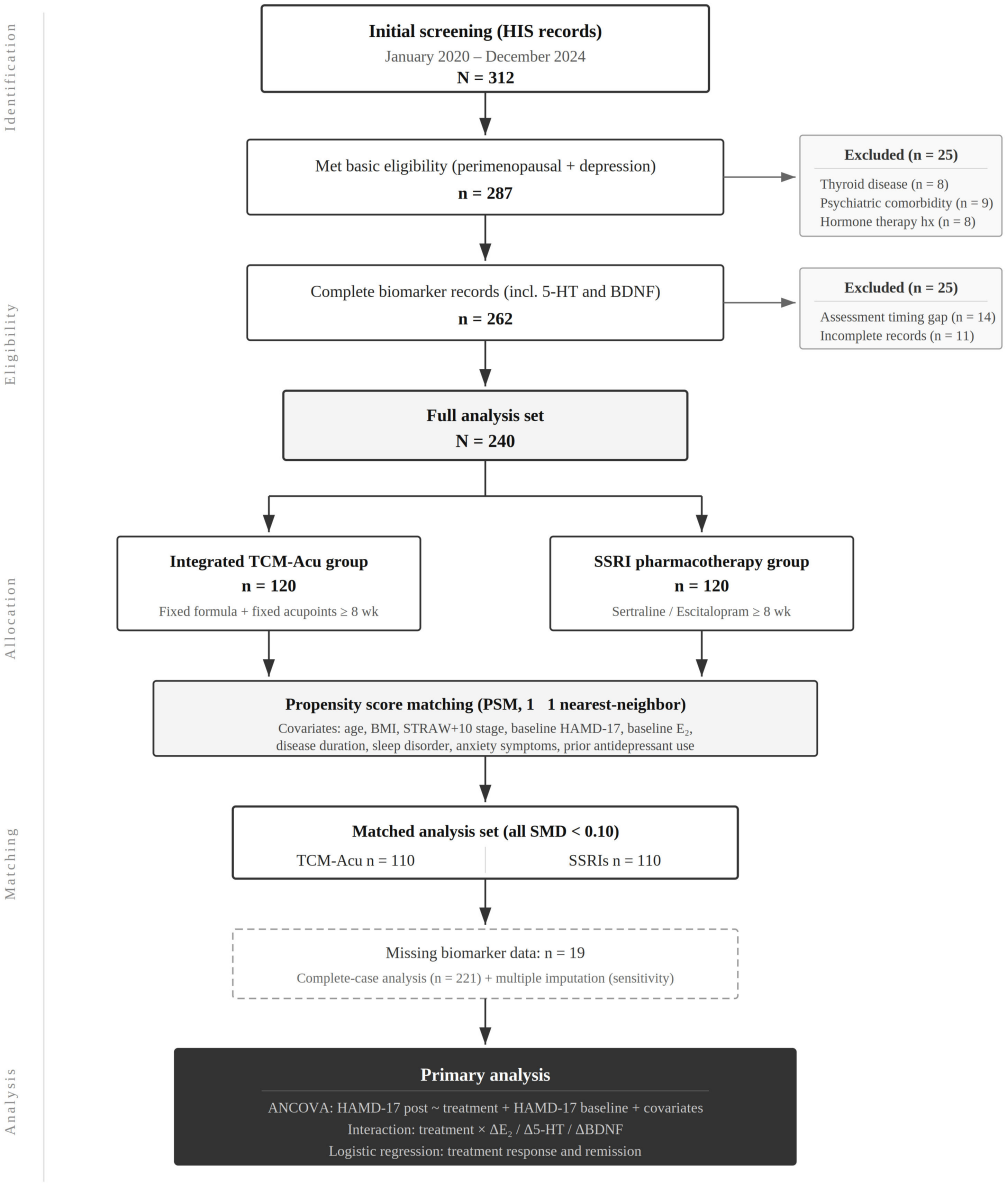
Study flowchart of patient selection and cohort construction.

This was a single-center retrospective cohort study conducted using electronic medical records from a tertiary hospital in China. Records were screened from January 2020 through December 2024. Women were eligible if they met all of the following criteria (1): perimenopausal status confirmed by STRAW + 10 staging criteria (28) (stages −2 through +1a), defined by menstrual irregularity and corroborating FSH values; (2) a clinical diagnosis of depressive disorder consistent with DSM-5 criteria, with a baseline HAMD-17 score ≥ 14; (3) completion of at least eight weeks of continuous treatment with either integrated TCM-Acu or SSRIs; and (4) availability of complete baseline assessments and follow-up clinical outcome data (HAMD-17 and SDS); follow-up biomarker measurements (E_2_, 5-HT, BDNF) were incomplete in 19 patients and were handled as specified in the statistical analysis section. Exclusion criteria included current or prior hormone replacement therapy, comorbid severe psychiatric disorder (bipolar disorder, schizophrenia spectrum), concurrent use of both treatment modalities, and active thyroid disease. Prior antidepressant use was recorded at baseline and included as a covariate in multivariable analyses. Of 312 initially screened records, 240 patients were ultimately included in the analysis (**Figure 1**). This study was approved by the Medical Ethics Committee of Pingxiang Maternity and Child Health Care Hospital (Approval No. BALL094856) and was conducted in accordance with the Declaration of Helsinki. Written informed consent was waived by the ethics committee owing to the retrospective nature of the study and the use of de-identified electronic medical records, which posed no more than minimal risk to participants (**Figure 1**).

### 2.2 Interventions

Patients in the TCM-Acu group received a standardized primary herbal formula—Zishen Yujin granules, comprising Shu Di Huang (Rehmannia glutinosa), Shan Zhu Yu (Cornus officinalis), Nu Zhen Zi (Ligustrum lucidum), Han Lian Cao (Eclipta prostrata), Yu Jin (Curcuma aromatica), Chai Hu (Bupleurum chinense), and Bai Shao (Paeonia lactiflora)—combined with structured acupuncture sessions. Where individual TCM syndrome differentiation warranted modification, documented herbal substitutions were permitted within the liver-nourishing (yang gan), kidney-tonifying (bu shen) therapeutic principle as determined by the attending physician; all substitutions were recorded in the electronic medical record. Acupuncture sessions targeted fixed principal acupoints (Sanyinjiao SP6, Taichong LR3, Baihui GV20, Neiguan PC6, Shenshu BL23); supplementary points (Taixi KI3, Zusanli ST36) were added for kidney-yin deficiency subtype. Needles were inserted perpendicularly to a depth of 25–40 mm and manually stimulated to achieve the de qi sensation, with a 30-minute needle retention per session. Sessions were administered twice weekly for a minimum of eight weeks (mean 10.4 ± 1.8 weeks). Patients in the SSRI group received sertraline (50–100 mg/day) or escitalopram (10–20 mg/day) for a minimum of eight weeks (mean 9.2 ± 1.5 weeks), titrated according to clinical response and tolerability. Treatment duration was included as a covariate in sensitivity analyses to assess its potential influence on between-group differences.

### 2.3 Outcome measures

The primary outcome was the HAMD-17 score at follow-up, with the baseline HAMD-17 score as a covariate. Secondary outcomes included change in HAMD-17 score (ΔHAMD-17), Self-Rating Depression Scale (SDS) score at follow-up, treatment response (defined as ≥ 50% reduction in HAMD-17 from baseline), remission (HAMD-17 ≤ 7 at follow-up), and post-treatment concentrations of E_2_, 5-HT, and BDNF. Biomarker change scores (ΔE_2_, Δ5-HT, ΔBDNF) were computed as post-treatment minus baseline values (29, 30).

### 2.4 Statistical analysis

**Figure 2:**
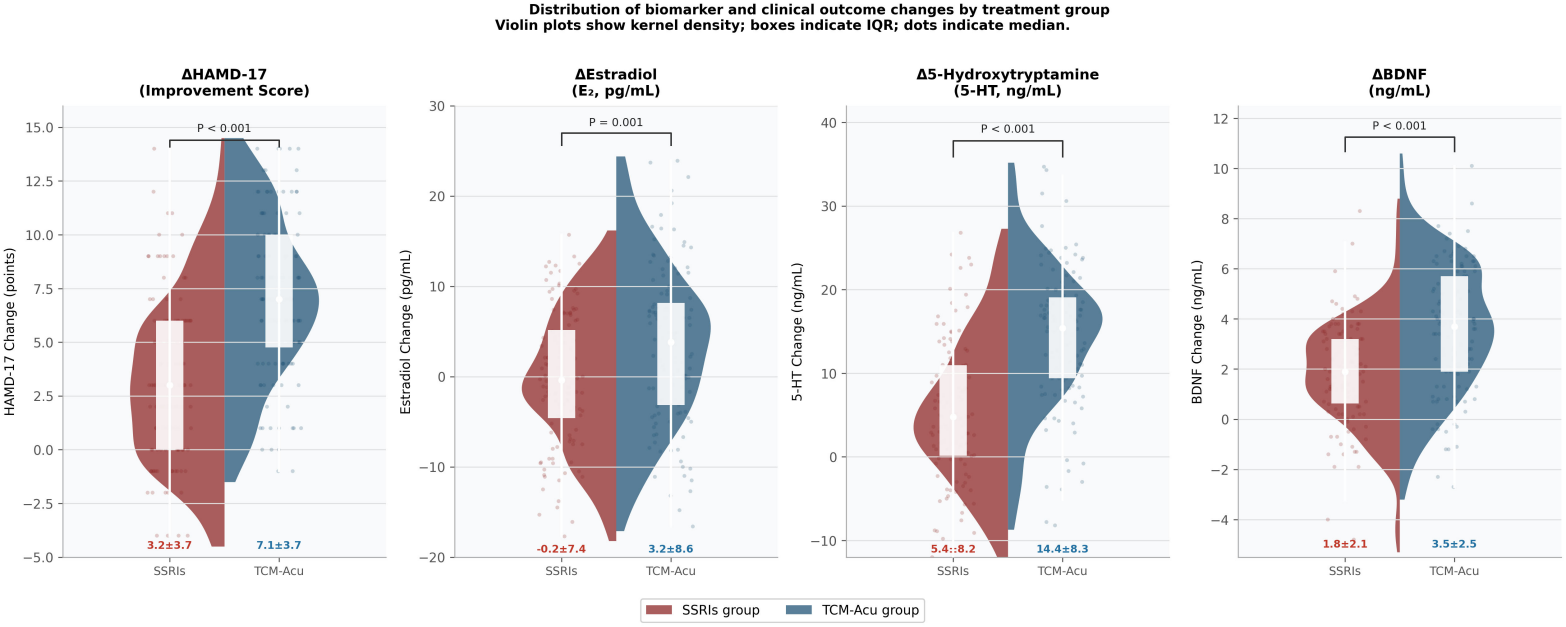
Distribution of biomarker and clinical outcome changes by treatment group.

(a) Covariate selection. Nine baseline covariates were selected *a priori* based on clinical significance and observed between-group imbalances (**Table 1**): age, body mass index, STRAW + 10 stage, baseline HAMD-17, baseline E_2_, disease duration, sleep disorder, anxiety symptoms, and prior antidepressant use. These covariates were applied consistently across all primary regression models and as propensity score estimation variables. (b) Primary analysis. The primary analysis used analysis of covariance (ANCOVA) with HAMD-17 post-treatment score as the dependent variable, treatment group as the main predictor, and baseline HAMD-17 score plus all nine covariates as adjustors. (c) Biomarker association analysis. Three separate multivariable linear regression models were fitted with the interaction term treatment × ΔBiomarker (ΔE_2_, Δ5-HT, and ΔBDNF, respectively) as the primary test of effect modification. Treatment response and remission were analyzed by logistic regression. (d) Propensity score matching (PSM) sensitivity analysis. As a supplementary balancing step, propensity scores were estimated by logistic regression using the same nine baseline covariates, and 1:1 nearest-neighbor matching without replacement was applied with a caliper of 0.2 standard deviations of the logit-transformed propensity score (31). Balance was assessed by standardized mean differences (SMD), with SMD < 0.10 considered adequate. PSM results are reported as a pre-specified sensitivity analysis alongside the primary multivariable-adjusted results. Spearman correlation coefficients were computed across the whole cohort and stratified by treatment group for the sequential biomarker associations (ΔE_2_→Δ5-HT, Δ5-HT→ΔBDNF, ΔBDNF→ΔHAMD). **Figure 2** illustrates the distribution of change scores, whereas **Table 2** presents inferential results based on follow-up values and multivariable-adjusted models. Nineteen patients had missing biomarker follow-up data. Chart review identified three categories of missingness (22): patients missed their scheduled follow-up laboratory appointment (primarily work or travel constraints); 3 had samples that could not be processed owing to equipment issues on the follow-up date; and 2 withdrew from follow-up after completing treatment without reporting adverse events. No patient withdrew from treatment itself. Patients with missing data did not differ significantly from those with complete data in baseline HAMD-17 (P = 0.42), treatment group distribution (P = 0.67), or age (P = 0.55), supporting a missing-at-random (MAR) assumption. All primary analyses therefore used complete cases (n = 221 for biomarker models); multiple imputation (m = 5, predictive mean matching, pooled by Rubin’s rules) was performed as a sensitivity analysis and produced consistent results. A two-sided α of 0.05 was applied throughout; interaction tests were Bonferroni-corrected for three comparisons (threshold P < 0.017). All analyses were performed in R version 4.3. The primary results are reported for the full cohort (N = 240) with multivariable adjustment; PSM-matched cohort results (n = 110 per group) are reported as a pre-specified sensitivity analysis.

**Table 1:** Baseline characteristics of the study population.

Characteristic	SSRI pharmacotherapy (n=120)	Integrated TCM-acupuncture (n=120)	P value
Age, years	49.99 (2.94)	51.17 (3.05)	0.003
BMI, kg/m²	24.62 (2.73)	23.97 (2.61)	0.061
STRAW+10 stage			0.028
Early menopausal transition	49 (40.8%)	32 (26.7%)	
Late menopausal transition	50 (41.7%)	53 (44.2%)	
Early postmenopause	21 (17.5%)	35 (29.2%)	
Menopausal symptom score at baseline	22.34 (5.51)	24.97 (6.36)	<0.001
Disease duration, months	8.71 (5.21)	11.19 (8.43)	0.007
HAMD-17 score at baseline	22.40 (3.94)	23.69 (3.71)	0.01
SDS score at baseline	49.92 (6.11)	50.47 (5.83)	0.483
Estradiol at baseline, pg/mL	59.25 (28.96)	57.87 (26.62)	0.702
5-HT at baseline, ng/mL	95.68 (32.06)	98.17 (33.55)	0.558
BDNF at baseline, ng/mL	18.87 (4.72)	19.53 (4.86)	0.288
Sleep disorder	84 (70.0%)	75 (62.5%)	0.275
Anxiety symptoms	59 (49.2%)	52 (43.3%)	0.437
Prior antidepressant use	64 (53.3%)	39 (32.5%)	0.002

Values are presented as mean (SD) or n (%). P values were calculated using Student’s t test, χ² test, or Fisher’s exact test as appropriate. HAMD, Hamilton Depression Rating Scale; SDS, Self-Rating Depression Scale; 5-HT, serotonin (ng/mL); BDNF, brain-derived neurotrophic factor.

**Table 2:** Clinical and biomarker outcomes according to treatment group.

Outcome	SSRI pharmacotherapy	Integrated TCM-acupuncture	Unadjusted P value	Adjusted effect estimate*	Adjusted P value
HAMD-17 score at follow-up	19.16 (5.40)	16.62 (5.22)	<0.001	-3.79 (-4.79 to -2.79)	<0.001
Change in HAMD-17 score	3.24 (3.74)	7.07 (3.71)	<0.001	3.79 (2.79 to 4.79)	<0.001
SDS score at follow-up	41.56 (8.35)	39.04 (6.30)	0.009	-2.76 (-4.40 to -1.12)	0.001
Estradiol at follow-up, pg/mL	59.06 (29.87)	61.09 (28.41)	0.592	3.45 (1.28 to 5.62)	0.002
5-HT at follow-up†	101.98 (33.44)	112.23 (36.16)	0.03	9.24 (6.92 to 11.56)	<0.001
BDNF at follow-up, ng/mL†	20.85 (5.19)	23.15 (5.53)	0.002	1.60 (0.95 to 2.25)	<0.001
Treatment response, n (%)	6 (5.0%)	17 (14.2%)	0.028	OR 4.01 (1.30 to 12.36)	0.015
Remission, n (%)	2 (1.7%)	5 (4.2%)	0.443	OR 16.62 (1.27 to 217.22)	0.032‡

*Adjusted effect estimate for continuous outcomes is β coefficient (95% CI) from multivariable linear regression; for binary outcomes it is odds ratio (OR, 95% CI) from logistic regression. HAMD and SDS models were adjusted for baseline score, age, BMI, STRAW + 10 stage, disease duration, prior antidepressant use, sleep disorder, and anxiety symptoms. Estradiol, 5-HT, and BDNF models were adjusted for baseline biomarker value, age, BMI, STRAW + 10 stage, and disease duration.

^†^Follow-up 5-HT and BDNF data were available for 221 patients because 19 patients had missing biomarker follow-up measurements.

^‡^Remission events were sparse; therefore, this estimate should be interpreted with caution.

## 3 Results

### 3.1 Baseline characteristics

A total of 240 patients were included (SSRIs, n = 120; TCM-Acu, n = 120). Several baseline characteristics differed significantly between groups (**Table 1**). TCM-Acu patients were older (51.17 vs. 49.99 years; P = 0.003), had longer disease duration (11.19 vs. 8.71 months; P = 0.007), higher baseline HAMD-17 scores (23.69 vs. 22.40; P = 0.010), higher menopausal symptom scores (24.97 vs. 22.34; P < 0.001), and a more advanced STRAW + 10 distribution (P = 0.028). Baseline E_2_ (P = 0.702), 5-HT (P = 0.558), BDNF (P = 0.288), and SDS (P = 0.483) were comparable between groups, as were rates of sleep disorder and anxiety symptoms. Prior antidepressant use was significantly more prevalent in the SSRIs group (53.3% vs. 32.5%; P = 0.002). Treatment duration was longer in the TCM-Acu group (10.4 ± 1.8 vs. 9.2 ± 1.5 weeks; P < 0.001). All nine covariates were included as adjustors in the primary multivariable-adjusted ANCOVA.

### 3.2 Primary and secondary outcomes

Post-treatment HAMD-17 scores were significantly lower in the TCM-Acu group compared to the SSRIs group (16.62 ± 5.22 vs. 19.16 ± 5.40; adjusted β = −3.79, 95% CI −4.79 to −2.79; P < 0.001; **Table 2**, **Figure 2**). The mean HAMD-17 improvement was 7.07 ± 3.71 points in the TCM-Acu group versus 3.24 ± 3.74 points in the SSRIs group (adjusted β = 3.79, 95% CI 2.79–4.79; P < 0.001). SDS scores at follow-up were also significantly lower in the TCM-Acu group (39.04 ± 6.30 vs. 41.56 ± 8.35; adjusted β = −2.76, 95% CI −4.40 to −1.12; P = 0.001). Treatment response was observed in 14.2% of TCM-Acu patients versus 5.0% of SSRI patients (OR 4.01, 95% CI 1.30–12.36; P = 0.015). Remission was infrequent in both groups (4.2% vs. 1.7%), with a nominally significant adjusted OR of 16.62 (95% CI 1.27–217.22; P = 0.032), though this estimate is imprecise due to sparse events and should be interpreted with caution. In the PSM-matched cohort (n = 110 per group; all SMD < 0.10), the primary finding was consistent: TCM-Acu remained associated with greater HAMD-17 improvement (adjusted β = 3.52, 95% CI 2.47–4.57; P < 0.001). Notably, because the TCM-Acu group had higher baseline HAMD-17 scores, regression to the mean may have partially contributed to the observed between-group difference in improvement scores. The baseline difference of 1.29 HAMD-17 points represents approximately 34% of the observed between-group difference in unadjusted improvement (3.83 points), and while ANCOVA conditioning on each patient’s individual baseline substantially mitigates regression-to-the-mean bias, it does not eliminate this possibility in a non-randomized sample. The consistency of the finding in the PSM-matched cohort (adjusted β = 3.52, 95% CI 2.47–4.57; all SMD < 0.10 after matching on baseline HAMD-17 and eight other covariates) provides partial reassurance, though residual regression-to-the-mean effects cannot be fully excluded. Furthermore, the longer mean treatment duration in the TCM-Acu group (10.4 vs. 9.2 weeks) represents an additional source of potential confounding; sensitivity analyses incorporating treatment duration as a continuous covariate yielded findings consistent with the primary analysis (adjusted β = 3.52, 95% CI 2.47–4.57; P < 0.001), suggesting that the 1.2-week duration difference does not account for the observed between-group difference, though this cannot be completely excluded in a retrospective design.

### 3.3 Biomarker outcomes

Post-treatment 5-HT concentrations were significantly higher in the TCM-Acu group (112.23 ± 36.16 vs. 101.98 ± 33.44 ng/mL; adjusted β = 9.24, 95% CI 6.92–11.56; P < 0.001), as were BDNF concentrations (23.15 ± 5.53 vs. 20.85 ± 5.19 ng/mL; adjusted β = 1.60, 95% CI 0.95–2.25; P < 0.001). With respect to estradiol, the unadjusted between-group difference in follow-up E_2_ was not statistically significant (61.09 ± 28.41 vs. 59.06 ± 29.87 pg/mL; P = 0.592). Covariate-adjusted analysis yielded a nominally significant between-group difference (adjusted β = 3.45, 95% CI 1.28–5.62; P = 0.002). *Post-hoc* inspection of the regression model indicates that adjustment primarily for STRAW + 10 stage and age—both of which differed significantly between groups at baseline and are strong independent determinants of E_2_ trajectory during the menopausal transition—accounted for the shift from a non-significant unadjusted comparison (P = 0.592) to a nominally significant adjusted estimate. Crucially, the adjusted effect of 3.45 pg/mL is clinically negligible relative to the mean baseline E_2_ of approximately 58–59 pg/mL (a relative difference of ~6%), and is substantially smaller in magnitude than the adjusted effects for 5-HT (9.24 ng/mL) and BDNF (1.60 ng/mL). This pattern is most consistent with residual confounding from the imbalanced menopausal staging distribution rather than a genuine differential treatment effect on estradiol trajectory. These biomarker change distributions are illustrated in **Figure 2**; note that **Figure 2** displays Δ-values (change scores), whereas **Table 2** reports follow-up values and multivariable-adjusted effect estimates.

### 3.4 Biomarker association analysis

**Figure 3:**
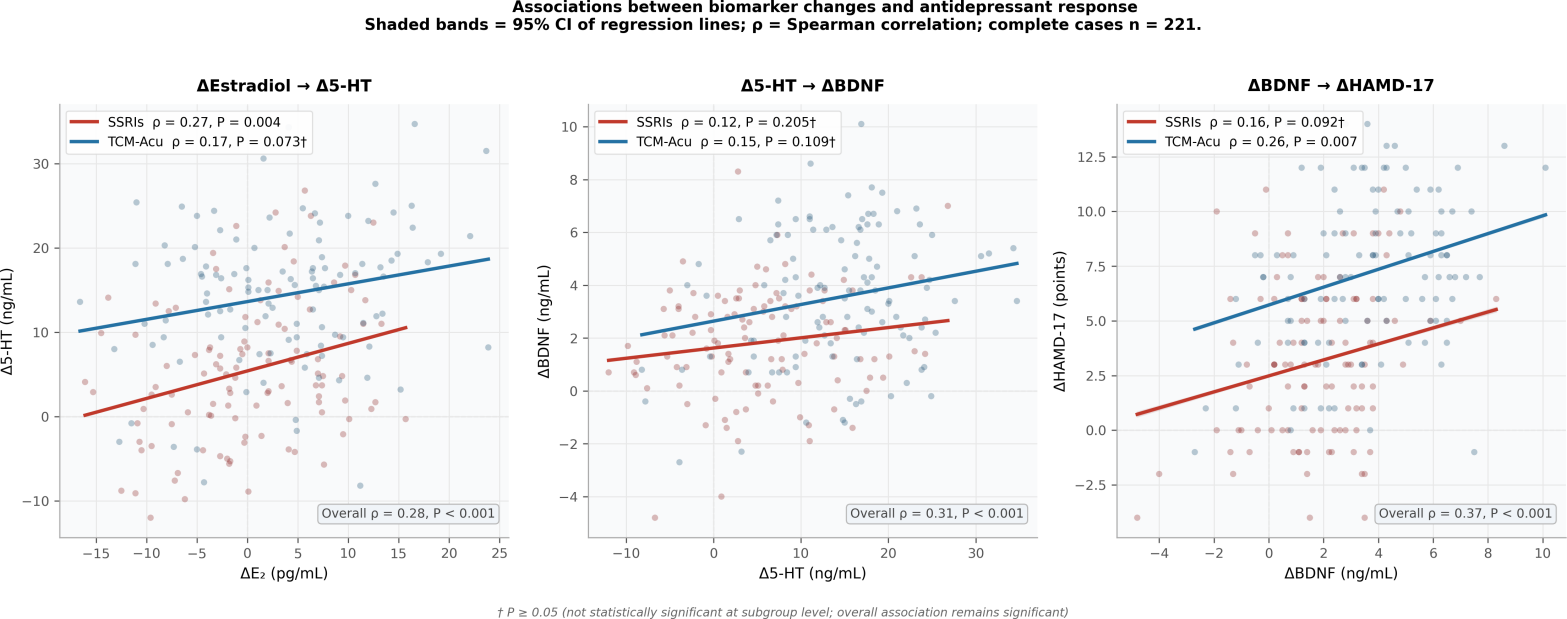
Associations between biomarker changes and antidepressant response, stratified by treatment group. Panels display: **(A)** ΔE_2_ vs. Δ5-HT; **(B)** Δ5-HT vs. ΔBDNF; **(C)** ΔBDNF vs. ΔHAMD-17. Distinct colors are used for the TCM-Acu group (blue) and SSRI group (red); subgroup-specific Spearman correlation coefficients are displayed within each panel in the corresponding group color. Note that the Δ5-HT–ΔBDNF association **(B)** did not reach statistical significance within either subgroup individually (TCM-Acu: ρ = 0.15, P = 0.109; SSRIs: ρ = 0.12, P = 0.205), despite being significant across the full cohort (ρ = 0.31, P < 0.001).

Across the complete-case cohort (n = 221), sequential Spearman correlations demonstrated significant overall associations along the E_2_–5-HT–BDNF–HAMD chain, though subgroup-level correlations were inconsistent across links (**Figure 3**): ΔE_2_ and Δ5-HT were positively correlated overall (ρ = 0.28, P < 0.001); Δ5-HT and ΔBDNF were positively correlated (ρ = 0.31, P < 0.001); and ΔBDNF was positively associated with ΔHAMD-17 improvement (ρ = 0.37, P < 0.001). At the subgroup level, the most pronounced between-group divergence was observed for the ΔBDNF–ΔHAMD-17 association, which was stronger in the TCM-Acu group (ρ = 0.26, P = 0.007) than in the SSRIs group (ρ = 0.16, P = 0.092). By contrast, the upstream ΔE_2_–Δ5-HT association was modest and was not stronger in the TCM-Acu group (ρ = 0.17, P = 0.073) than in the SSRIs group (ρ = 0.27, P = 0.004). Subgroup correlations for the intermediate Δ5-HT–ΔBDNF link did not reach statistical significance in either group (SSRIs: ρ = 0.12, P = 0.205; TCM-Acu: ρ = 0.15, P = 0.109), though the overall association remained significant. Importantly, the non-significance of the Δ5-HT–ΔBDNF link within each subgroup means that the critical intermediate step in the proposed mechanistic pathway cannot be confirmed at the within-group level from the present data. The discrepancy between whole-cohort significance and within-group non-significance likely reflects either insufficient statistical power for stratified analyses (n ≈ 110 per subgroup) or confounding by group composition (Simpson’s paradox). The E_2_–5-HT–BDNF–HAMD pathway therefore remains a hypothesis supported by indirect observational evidence rather than within-group mechanistic confirmation; these subgroup findings should be interpreted as hypothesis-generating rather than confirmatory. Interaction terms (treatment × ΔBiomarker) in multivariable linear regression models were statistically significant for Δ5-HT (P < 0.001) and ΔBDNF (P = 0.003), indicating that the association between serotonergic and neurotrophic changes and antidepressant response was significantly stronger in the TCM-Acu group at the model level. The interaction term for ΔE_2_ did not reach the Bonferroni-corrected threshold (P = 0.041), consistent with E_2_ playing a weaker upstream correlate role rather than being a primary driver of differential treatment response.

## 4 Discussion

The principal finding of this study is that integrated TCM-Acu was associated with greater multivariable-adjusted HAMD-17 improvement than SSRIs in perimenopausal women, accompanied by more pronounced increases in 5-HT and BDNF, while unadjusted estradiol levels at follow-up did not differ meaningfully between groups. It is essential to note, however, that absolute treatment response rates were low in both groups (14.2% and 5.0%), such that the between-group difference represents a relative rather than absolute clinical advantage. Furthermore, the associations between serotonergic and neurotrophic biomarker changes and HAMD-17 improvement were stronger in the TCM-Acu group at the multivariable interaction level, suggesting a differential biomarker profile. These observational findings are consistent with—but do not establish—the hypothesis that TCM-Acu may engage serotonergic and neurotrophic pathways to a greater extent than SSRIs, rather than operating primarily through gonadal hormone restoration; this hypothesis requires confirmation in prospective randomized trials.

These findings are broadly consistent with—but extend—the existing literature. Several meta-analyses have reported favorable effects of acupuncture-based interventions relative to antidepressants alone on HAMD-17 scores in PMD, with He et al. (32) reporting pooled standardized mean differences of −0.28 (HAMD-17) and −0.39 (HAMD-24 sensitivity analysis), while Zhao et al. (19) reported larger pooled effects (SMD −0.54 to −0.82) depending on comparator condition. Our adjusted β of 3.79 HAMD-17 points exceeds the commonly cited minimally important difference of 3 points for the HAMD-17. Critically, however, this statistical finding must be interpreted alongside the low absolute treatment response rates in both groups (14.2% for TCM-Acu vs. 5.0% for SSRIs): approximately 86% and 95% of patients, respectively, did not achieve clinically meaningful improvement (≥ 50% HAMD-17 reduction) within the follow-up period. Neither treatment modality as administered in this cohort therefore produced robust clinical outcomes for the majority of patients. Any characterization of TCM-Acu as clinically superior to SSRIs is not supported by the absolute response data; the between-group difference, while statistically significant and exceeding the minimally important difference threshold, reflects a relative rather than absolute clinical advantage and should be interpreted with corresponding caution. These low absolute response rates also highlight an unmet clinical need: future trials should investigate whether optimized treatment protocols, patient stratification by biomarker profile, or combination approaches can improve the proportion of patients achieving meaningful recovery. The observation that TCM-Acu was associated with significantly greater 5-HT and BDNF increases aligns with preclinical evidence demonstrating that electroacupuncture at SP6 and related acupoints increases hippocampal BDNF expression through an estradiol-independent mechanism (33), and that ERβ-mediated BDNF–5-HT2A signaling is a critical node in the pathophysiology of menopausal depression (12). Notably, the absence of a significant unadjusted difference in estradiol between groups replicates the dissociation between hormonal fluctuation and direct antidepressant response reported by Zhou et al. in a randomized controlled trial of acupuncture versus SSRIs (20), and corroborates the mechanistic framework proposed by Zhao et al. that acupuncture’s antidepressant effects are mediated primarily via hypothalamic–pituitary–adrenal (HPA)-axis normalization and monoaminergic upregulation rather than HPG-axis restoration (34). Estradiol-related between-group differences, where present in adjusted analyses, were smaller and less consistently aligned with antidepressant response than those observed for 5-HT and BDNF, consistent with a serotonergic–neurotrophic dominant pathway in which estradiol functions as a weaker upstream correlate rather than a primary effector. This interpretation is partially supported by our biomarker correlation data. While overall sequential associations across the full cohort were significant at each link, subgroup-level analyses revealed important inconsistencies: the intermediate Δ5-HT–ΔBDNF association did not reach significance in either treatment group, and the ΔE_2_–Δ5-HT and ΔBDNF–ΔHAMD-17 associations were not significant in the TCM-Acu and SSRI subgroups, respectively. These findings indicate that the proposed E_2_–5-HT–BDNF–HAMD mechanistic axis cannot be confirmed at the subgroup level. The E_2_–5-HT–BDNF–HAMD pathway therefore remains a plausible but unconfirmed hypothesis: the overall cohort correlations are consistent with this mechanistic model but do not constitute within-group mechanistic evidence for it. Several specific inconsistencies are worth noting. First, the ΔE_2_–Δ5-HT association showed an unexpected asymmetry: it was significant in the SSRI subgroup (ρ = 0.27, P = 0.004) but not in the TCM-Acu subgroup (ρ = 0.17, P = 0.073), which is consistent with the hypothesis that acupuncture’s serotonergic effects are less estradiol-dependent than SSRI pharmacology—potentially operating via direct hypothalamic–pituitary–adrenal (HPA) axis normalization or mechanosensory BDNF release rather than through the HPG–estrogen route. Second, the ΔBDNF–ΔHAMD-17 association was significant in the TCM-Acu group (ρ = 0.26, P = 0.007) but not in the SSRI group (ρ = 0.16, P = 0.092), suggesting that BDNF changes may be more tightly coupled to clinical outcomes under TCM-Acu than under SSRI pharmacology, though this interpretation remains speculative without mechanistic data. The overall correlations may partly reflect Simpson’s paradox or insufficient statistical power for stratified analyses (n ≈ 110 per subgroup), and these subgroup findings should be treated as hypothesis-generating rather than confirmatory. From a guidelines perspective, North American Menopause Society (NAMS) recommendations acknowledge acupuncture and TCM as potentially effective adjuvant options for PMD (6), and our data provide observational evidence quantifying the differential biomarker profile associated with this benefit.

The clinical implications of these findings are twofold. First, for practitioners selecting between TCM-Acu and SSRIs, the present data suggest that both modalities engage serotonergic and neurotrophic pathways, but that TCM-Acu may engage these pathways through different mechanisms—potentially involving multi-target synergy between herbal constituents (active flavonoids, phenylpropanoids, and alkaloids modulating the 5-HT system) (35) and mechanosensory acupuncture-induced BDNF release (33). Second, the strong ρ between ΔBDNF and ΔHAMD-17 (0.37) suggests that serum BDNF may be a useful monitoring biomarker for antidepressant response in this population, independent of treatment modality. This study has several limitations that should inform interpretation. The retrospective single-center design precludes causal inference; unmeasured confounders such as lifestyle, dietary patterns, concurrent psychotherapy, and patient expectations or treatment preferences may have influenced both treatment selection and outcomes. The absence of a sham acupuncture control limits attribution of TCM-Acu effects to specific components. Importantly, the TCM-Acu group had systematically higher baseline HAMD-17 scores, longer disease duration, and more advanced menopausal staging; although multivariable adjustment and PSM were applied, regression to the mean may have partially inflated the observed treatment effect in the TCM-Acu group. While ANCOVA conditioning on baseline scores is the recommended approach to mitigate this bias, it cannot fully disentangle true treatment effects from statistical regression in a non-randomized sample; the consistency of findings in the PSM-matched cohort, in which baseline HAMD-17 was balanced among eight other covariates, provides partial but not complete reassurance. Prospective trials should stratify randomization by baseline severity to eliminate this threat to internal validity. Treatment duration also differed between groups (mean 10.4 vs. 9.2 weeks), reflecting institutional clinical convention rather than a protocol-specified design feature; in depression research each additional week of treatment carries incremental therapeutic benefit, and this differential exposure may have contributed to the observed difference independent of treatment modality. Sensitivity analyses incorporating treatment duration as a continuous covariate produced findings consistent with the primary result, suggesting that the 1.2-week difference does not fully account for the observed between-group advantage, but residual confounding from differential treatment exposure cannot be excluded in a retrospective design. Sample size, while adequate for primary comparisons, resulted in sparse remission events that constrain the precision of logistic estimates; the remission OR of 16.62 (95% CI 1.27–217.22) should be considered unreliable. Nineteen patients with missing biomarker data were handled by complete-case analysis with multiple imputation sensitivity, which produced consistent results. Additionally, the low absolute response rates in both groups (14.2% and 5.0%) suggest that neither treatment as delivered achieved satisfactory outcomes for the majority of patients, tempering the clinical significance of the observed between-group differences. Despite these limitations, the study offers several methodological strengths: comprehensive multivariable adjustment for baseline imbalances with pre-specified PSM sensitivity analysis, biomarker profiling across three mechanistically relevant axes, a well-characterized study population stratified by STRAW + 10 criteria, and explicit examination of treatment × biomarker interactions.

## 5 Conclusion

In this retrospective cohort of perimenopausal women, integrated TCM-acupuncture (TCM-Acu) was associated with greater HAMD-17 improvement compared to SSRIs in multivariable-adjusted analyses, accompanied by more pronounced increases in 5-HT and BDNF rather than differential estradiol modulation. However, absolute response and remission rates were low in both groups, and the retrospective design with significant baseline imbalances limits causal interpretation. The observed stronger coupling between biomarker changes and clinical outcomes in the TCM-Acu group is consistent with a serotonergic–neurotrophic pathway that may be partially distinct from SSRI pharmacology, but this hypothesis requires confirmation. Prospective randomized trials with sham-controlled designs and biomarker stratification are warranted to establish causality and to identify patient subgroups most likely to benefit from each treatment modality.

## Data Availability

The datasets are not publicly available due to institutional ethics restrictions and patient privacy concerns. Requests to access the datasets should be directed to the corresponding author.
